# Increased Levels of Serum Uric Acid among Ex-smokers

**DOI:** 10.2188/jea.JE2006332

**Published:** 2008-05-29

**Authors:** Masako Tomita, Shoichi Mizuno, Kazuhiko Yokota

**Affiliations:** 1 JR East Health Promotion Center; 2 Tokyo Metropolitan Institute of Gerontology

**Keywords:** Smoking Cessation, Uric Acid, Body Mass Index, Alcohol Drinking

## Abstract

**Background:**

It remains unclear whether serum uric acid level increases after the cessation of smoking.

**Methods:**

In 2000, we conducted a cross-sectional study on the effects of smoking cessation on serum uric acid levels by analyzing the results of annual health check-ups in the Japanese male working population (n = 16,642).

**Results:**

The serum uric acid level (6.18 mg/dL) was the highest in ex-smokers, followed by that in never-smokers (6.10 mg/dL) and that in current smokers (5.98 mg/dL). Ex-smokers weighed 0.6 kg more than the never-smokers and 1.5 kg more than the current smokers. The frequency of alcohol intake was closely correlated to the smoking habits. The serum uric acid levels declined in all groups, after adjustments for age, body mass index, and alcohol intake, though the levels in ex-smokers were 0.2 mg/dL higher than those in current smokers.

**Conclusion:**

The results suggested that alcohol intake contributed considerably to the serum uric acid levels and that smoking itself may have suppressed these levels via metabolic effects or the action of superoxides.

## INTRODUCTION

Chronic smokers have been reported to have an increased risk of cancer and cardiovascular diseases (CVDs). Cigarette smoke contains superoxides and other reactive oxygen species, and it has been hypothesized that some of the adverse effects of smoking may result from oxidative damage to endothelial cells.^1^ An *in vitro* biological study demonstrated the antioxidative role of serum uric acid (UA) in protection against DNA damage.^2^ *In vivo*, superoxides and other reactive oxygen species present in cigarette smoke may decrease the serum levels of antioxidants, including UA.^3^^,^^4^ On the other hand, hyperuricemia is strongly associated with the relative risk (RR) of death from CVDs,^5^^,^^6^ although the precise role of UA in the development of CVDs has not yet been clarified.

Our previous research revealed that smoking was negatively correlated with body mass index (BMI), serum total cholesterol, and UA.^7^ We also demonstrated that ex-smokers showed higher UA levels than those of current smokers or never-smokers.^7^ At that time (in 1989), the proportion of smokers and ex-smokers among our subjects was 70% and 10%, respectively, and our main aim was to study the effects of smoking on physical and laboratory data. Recently, the incidence of smoking among Japanese males has been gradually decreasing, especially after the enforcement of the Health Promotion Law in 2004 (Tobacco Free Japan 2005, http://www.tobaccofree.jp). In our current study population, the proportion of ex-smokers approached 24%, enabling the estimation of their health indexes and being an interesting theme.

This study aimed to quantitatively evaluate the increase in UA levels in ex-smokers and to propose a hypothesis taking into account the combination of excessive weight and excessive alcohol consumption, for lowering increased UA levels to within the normal range.

## METHODS

This cross-sectional study was conducted in 2000, using annual health check-up data from 16,642 Japanese male railroad workers, aged 35-59 years. The participation rate in annual health check-ups in this population was as high as 97%.

The health check-up items included a blood chemistry test, including tests for UA; height and weight measurements; and questionnaires regarding the subjects’ lifestyles. Based on their smoking habits, the subjects were classified into never-smokers (n=3143, 18.9%), current smokers (n=9476, 56.9%), and ex-smokers (n=4023, 24.2%). Alcohol intake was classified in terms of days per week. The proportion of ex-smokers increased with age, reaching approximately 30% among those in their 50s.

The UA levels (mg/dL) were examined with a Hitachi 7600-110 analyzer, using the peroxidase assay.^8^ The software SPSS^®^ was used to determine the frequencies, group means, and regression coefficients. The UA levels were compared among subjects classified according to their smoking habits, by multivariate linear models with adjustments such as age to its mean 47.5, BMI to 23.6 kg/m^2^, and drinking status to non-drinking. This study was approved by the JR East Health Promotion Center Ethical Committee for Epidemiological Studies.

## RESULTS

The UA level was the highest in ex-smokers, followed by that in never-smokers and that in current smokers (Table 1). The UA level in ex-smokers was 0.08 mg/dL higher than that in never-smokers and 0.2 mg/dL higher than that in current smokers (*P* < 0.001).

**Table 1.** Characteristics of subjects according to the smoking category.

Item	Ex-smokers	Smokers	Never-smokers
No. of subjects	4023	9476	3143
Age (year)	48.6* (5.9)	47.0 (5.9)	47.7 (6.3)
Serum uric acid (mg/dL)	6.18* (1.36)	5.98 (1.32)	6.10 (1.32)
Body weight (kg)	67.3* (8.9)	65.8 (9.8)	66.7 (9.4)
Alcohol intake (d/wk)	3.6* (2.3)	3.4 (2.3)	2.9 (2.4)
BMI (kg/m^2^)	23.9* (2.7)	23.3 (3.1)	23.8 (3.0)

Mean (standard deviation)

* : *P* < 0.001 (compared to smokers)

BMI : body mass index

The ex-smokers weighed 0.6 kg more than the never-smokers and 1.5 kg more than the current smokers (*P* < 0.001). Moreover, the BMI progressively decreased among ex-smokers, never-smokers, and current smokers. The age-adjusted regression coefficient of UA and BMI was 0.11 (*P* < 0.01) among never-smokers (UA = 0.11 x (BMI - 23.6) -0.016 x (age - 47.5) + 4.64).

The frequency of alcohol intake was the highest among ex-smokers, followed by the frequencies among current smokers and never-smokers. The alcohol intake frequency was closely correlated to the smoking habits (*P* < 0.001).

The UA level progressively decreased among the ex-smokers, never-smokers, and current smokers when adjusted for age and BMI. The same trend was observed (Table 2) when adjustments for age, BMI, and alcohol intake were performed. The UA level among daily drinkers was 6.41 mg/dL, which is 0.42 mg/dL higher (*P* < 0.001) than the level among non-drinkers, i.e., 5.99 mg/dL. The age-adjusted regression coefficient of UA and alcohol intake was 0.056 (*P* < 0.001) in never-smokers (UA = 0.056 x drink days - 0.022 x (age - 47.5) + 7.03).

**Table 2.** Adjusted means (standard errors) of serum uric acid levels according to smoking habits.

Item	Ex-smokers	Smokers	Never-smokers
Crude	6.18* (0.02)	5.98 (0.01)	6.10 (0.02)
Adjusted for age	6.21* (0.02)	5.97 (0.01)	6.11 (0.02)
Adjusted for age and BMI	6.18* (0.02)	5.99 (0.01)	6.08 (0.02)
Adjusted for age, BMI, and alcohol consumption	5.92* (0.03)	5.74 (0.02)	5.87 (0.03)

Mean (standard error)

* : *P* < 0.001 (compared to smokers)

BMI : body mass index

## DISCUSSION

To the best of our knowledge, this is the first study that examined the effects of smoking on the serum UA levels on a large scale, i.e., in more than 4,000 ex-smokers. The results revealed that the UA levels were the highest among ex-smokers, followed by those among the never-smokers and the current smokers. As our study has already reported, a significant association was observed among body weight, alcohol intake, and UA levels.^9^ When adjusted for age, BMI, and alcohol intake, the UA levels declined in the 3 groups, though the UA level in the ex-smokers remained 0.2 mg/dL higher than that in the current smokers. The results suggested a considerable contribution of alcohol intake to UA levels, and that smoking itself may have suppressed the UA level via metabolic effects or the action of superoxides. Subjects who had quit smoking (quitters) experienced a 1.5 kg increase in body weight. Subjects who gained weight after smoking cessation were expected to eventually return to their normal and/or regular weight; further, the benefits of smoking cessation were considered to surpass the ill effects of weight gain.^10^ Smoking habits were also associated with alcohol intake. Subjects who stop smoking may not necessarily stop drinking; on the contrary, they may increase their alcohol intake to compensate for their feeling of unease stemming from smoking cessation.

It was assumed that the body weight and frequency of alcohol intake changed after smoking cessation, and that these changes were closely related to the increase in the UA level among ex-smokers. Increase in the UA levels is a serious problem for ex-smokers who had high UA levels while smoking. To avoid this unfavorable increase in UA (in the range of 0.2 mg/dL) after quitting smoking, we investigated methods of reducing the increased UA levels. Our regression analysis revealed that the age-adjusted regression coefficient of UA and BMI was 0.11 for never-smokers. In overweight persons, the potential elevation in UA levels after smoking cessation may be prevented by decreasing the body weight.

Furthermore, our regression analysis revealed that the age-adjusted regression coefficient of UA and of the number of drinking days was 0.056 in never-smokers. The 0.2-mg/dL increase in the UA levels after smoking cessation may correspond to a 4-day/week frequency of alcohol consumption.

In conclusion, the present study suggested that smokers who drink frequently should decrease their alcohol intake after smoking cessation. Hyperuricemia after smoking cessation should be investigated, and health promotion should be undertaken to prevent excessive weight gain and maintain appropriate drinking habits in the at-risk population. Smoking cessation programs combined with suitable nutritional guidance should be investigated in interventional studies.
